# Mother-to-child transmission of hepatitis B: extent of knowledge of physicians and midwives in Eastern region of Ghana

**DOI:** 10.1186/s12889-016-3215-6

**Published:** 2016-07-11

**Authors:** Charles Ampong Adjei, Richard Asamoah, Fidelis Atibila, Gilbert Nachinab Ti-enkawol, Michael Ansah-Nyarko

**Affiliations:** Department of Nursing, Valley View University, Box AF 595, , Adentan, Accra, Ghana; University of Ghana Hospital, Legon, Accra, Ghana; Dormaa Presbyterian Hospital, Box 47, Dormaa Ahenkro, Ghana; Presbyterian Nurses’ Training College, Box 45, Bawku, Upper East Region Ghana; University of Ghana, Accra, Ghana

**Keywords:** Hepatitis B, Mother-to-child, Knowledge, Ghana, Physicians, Midwives

## Abstract

**Background:**

Mother -to -Child transmission of hepatitis B infection remains a major public health concern particularly in Africa. Adequate knowledge of physicians and midwives is crucial in averting most of the hepatitis B viral transmissions from mothers to their new-borns. However, there is a dearth of evidence on extent of knowledge of physicians and midwives in Ghana inspite of the increasing incidence of hepatitis B infection in the country. This study therefore assessed the knowledge level of physicians and midwives regarding Mother-to-Child transmission of hepatitis B in the Eastern region of Ghana.

**Methods:**

A Cross sectional survey was conducted between August to November, 2015 using semi-structured self-administered questionnaire. Study participants were recruited from five health facilities and their level of awareness and knowledge about Mother-to-Child transmission of hepatitis B were assessed. The level of statistical significance was set at 0.05 alpha level.

**Results:**

The findings showed that both physicians and midwives had good knowledge on Mother-to-Child transmission of hepatitis B infection. However, there were some knowledge gaps regarding effective hepatitis B prevention from mother to their newborns such as the use of hepatitis B vaccine and immunoglobulin. Additionally, 49.2 % (*n* = 62) of the participants had never attended any workshop on Mother-to-Child transmission of hepatitis B since completion of formal training.

**Conclusions:**

Developing appropriate periodic training programmes on current issues of hepatitis B for physicians and midwives in Eastern region will further enhance their knowledge. It is recommended that, further study examine if the knowledge of the respondents is translated into practice.

## Background

Hepatitis B viral (HBV) infection remains a global public health challenge accounting for a significant proportion of deaths attributable to cancer worldwide [1]. The global estimate indicates that about 240 million individuals are chronically infected with HBV and approximately 780,000 deaths occur from the consequences of HBV infection annually [2]. A recent report by the World Health Organisation shows that the majority of persons living with HBV infection reside in low and middle income countries including Sub-Saharan Africa (SSA) and Asia [3]. Ghana is one of the West African countries which is hyper endemic of HBV with a prevalence estimate of 15 % of adult population [4].

There is evidence that the probability of HBV infection progressing from acute to chronic stage depends on the age at which a person becomes infected [3, 5, 6]. Infection in early life has a much higher risk of becoming chronic than later in life. About 80–90 % of neonates, and 30–50 % of children develop chronic HBV infection when exposed to the virus [2]. Also, they stand a higher risk of developing chronic liver disease including hepatocellular carcinoma and cirrhosis of the liver than those infected as adults [3].

It is well documented that perinatal transmission is an important source of HBV infection to newborns [5, 7], and therefore, HBV positive pregnant women predispose their infants to serious risk of transmission during birth [8]. The huge contribution of about 50 % perinatal transmission in high endemic countries makes its crucial to implement standard protocols and interventions which target prevention of MTCT of HBV infection [9, 10].

Centers for Disease Control and Prevention (CDC) recommends routine screening of pregnant women during the ante-natal period to identify those who are positive for hepatitis B surface antigen (HBsAg) [8]. In addition, administration of Hepatitis B vaccine and Hepatitis B immunoglobulin (HBIG) to newborns of HBV infected mothers within the first 12 h of birth remains an effective intervention, averting about 90–95 % of MTCT of HBV infections [2, 8, 9]. However, these measures are not well implemented in many health facilities in Ghana. A study conducted in the Eastern region of Ghana found that newborns to HBV infected mothers are not given hepatitis B vaccine and HBIG at birth except the routine vaccination which are administered at 6, 10, and 14 weeks after birth [11]. This according to the study happened although pregnant women were screened for HBsAg in the health facilities [11]. This is a huge gap in Ghana’s response to the burden of hepatitis B infection particularly with the high prevalence of HBsAg (10.6 to 16 %) among pregnant women in the country [11, 12].

Several studies have assessed the level of knowledge of pregnant women on HBV infection and the evidence indicates gaps in their knowledge [13–15]. However, only few studies have assessed physician and midwives levels of knowledge on prevention of MTCT of HBV infection [16, 17]. A study conducted in China revealed that, majority of perinatal nurses including obstetrics, maternity, labour and intensive care unit nurses were unaware of the risk of newborns to chronic HBV infection which influenced their poor preventive practices [16].

In Ghana, there is a dearth of empirical evidence on the level of knowledge of midwives and physicians on MTCT of HBV infection. This study therefore aimed at establishing a baseline data on the subject. This is significant considering the fact that, in Ghana, physicians and midwives are the core staff that request hepatitis B screening during pregnancy and administer hepatitis B vaccine and immunoglobulin to newborns at birth. More so, these staff serve as the main source of hepatitis B-related information for most clients [18]. The findings of this study will contribute to programme design that will meet the learning needs of the professionals and inform policy.

## Methods

### Setting

Eastern region is the sixth largest region of Ghana. However, it is the third most populous region in the country, after Ashanti and Greater Accra region. It occupies an area of about 19,323 km^2^ representing 8.9 % of the total land area of Ghana. It has a population of 2,633,154 which makes up 10.7 % of the total population of Ghana [19]. Estimates of 50.8 % of the populace are females whereas 49.2 % are males. The region shares boarders with Volta region in the East, Greater Accra region in the south, Central region in the west and Ashanti region in the north [19].

The study was conducted in the main Regional Hospital and four district hospitals comprised of two mission and two Government health facilities. This was to allow for statistical comparison to find out if there are contextual differences in knowledge of mother to child transmission (MTCT) of HBV infection among participants.

### Study design, participants and sampling

A quantitative approach using a cross sectional survey was conducted between August and November, 2015. Physicians and midwives working in the Eastern regional hospital and four district hospitals including mission and government owned facilities were recruited for the study. Participants who were full time employees of the selected health facilities with atleast six (6) months working experience in midwifery or medicine were recruited. In addition, participants currently providing maternal and neonatal care who consented to participate in the study were selected. Physicians and midwives undergoing internship and national service were excluded from the study.

A census approach was employed to recruit the physicians. Census approach was used because the entire population of physicians in the selected facilities is small and therefore any physician who met the inclusion criteria and who consented to participate at the time of data collection was included. Oral messages were sent to physicians working in the selected facilities a week prior to data collection date to inform them about the study. On the said day of census, physicians were contacted individually in their consulting room for inclusion after obtaining their written informed consent.

However, a convenience sampling technique was used to select the midwives at both regional and district hospitals. Convenience sampling is a type of a non-probability sampling technique in which members of the target population are chosen based on their availability, accessibility and readiness to voluntarily participate in a study [20]. Verbal announcement was made a week prior to data collection to inform the midwives in their respective unit about the study. On the said date, midwives were contacted at their respective units of work including the antenatal, labour ward, lying-in ward, post-natal unit for inclusion after obtaining written informed consent from them. Using the total number of physicians and midwives in the facilities, proportional quota was used to determine the sample for each hospital.

### Sample size determination

Yamane (1967) [21] formula with a level of precision of 0.05 was used to determine the sample size for the study. In all, 130 questionnaires were administered and 126 of them were retrieved indicating a 97 % response rate.

### Study procedure and data collection

A semi-structured self- administered questionnaire in English language was administered to eligible participants. The questionnaire was designed by the research team based on literature review and its content validity was established through consultation with three experts in the field. Five physicians and ten midwives were first used to pre-test construct validity of the instrument and the items which were found to be ambiguous and difficult to understand were noted from the responses and corrected. In addition, their experts knowledge was considered. After the correction was done, 30 participants were further given the questionnaires to answer. Using Cronbach alpha to test the internal consistency, the instrument was found to be 0.76 indicating that the instrument is reliable. The questionnaire had three sections. The socio-demographic characteristics, assessment of awareness level of hepatitis B and extent of knowledge of MTCT of hepatitis B among study participants. Twelve (12) questions were asked on both awareness and knowledge. On the knowledge scale, a score ranging from 0–2 is considered poor, 3–5 fair and 6–9 is good. From the current data, the mean score for the sample is 6.10 and a standard deviation of 1.17. The highest possible score on the knowledge is 9.

### Data analysis

Data collected was analysed using Statistical Package of Social Science (IBM-SPSS) version 22.0. In analysing the data, descriptive statistic such as means, percentage and standard deviation were examined. Additionally, independent *t*-test was used to determine the differences in knowledge on MTCT between physicians and midwives. The difference in knowledge on MTCT and participant’s socio-demographic characteristics were analysed using Analysis of the variance (ANOVA). A *p*- value of 0.05 alpha level was considered statistically significant.

## Results

### Socio-demographic characteristics of participants

Overall, 126 participants were recruited for this study. Out of this, 39 (31.0 %) were physicians constituting a response rate of 76.5 % of the expected number of physicians working in the selected health facilities. In addition, 87 (69.0 %) of the participants were midwives. Furthermore, female participants were 100 (79.4 %) as compared to 26 males representing 20.6 %. Data was skewed toward females because only female midwives work in the study settings. Also, some of the physicians were females which added onto the already large number of female midwives. In addition 22 (17.5 %) of the participants were within the age category of 20-25years, 56 (44.4 %) within 26–30 years, 22 (17.5 %) within 31–35 years, 12 (9.5 %) within 36–40years, 1 representing 0.8 % was within 41–45years. Also, 3 (2.4 %) of the participants fell within the age bracket of 46–50 years and 10 (7.9 %) were 51 years and above. The type of health facility was also examined. Out of the 126 participants, 38 (30.2 %) were from Eastern Regional Government Hospital. Overall, 42 (33.3 %) participants were sampled and tested from the district government hospitals which include 24 (19 %) and 18 (14.3 %) from Oda Government and Nsawam Government hospital respectively. From the district mission hospital, 46 (36.5 %) participants were sampled and tested of which 26 (20.6 %) were from St. Joseph and 20 (15.9 %) were from St. Martins hospitals. Marital statuses, qualification, years of service and unit of work were examined. Summary of descriptive statistics are presented in Table 1 below.

**Table 1:** Summary of descriptive statistics of participants (*n* = 126)

Variables	Frequency	Percentage (%)
Profession		
• Midwives	87	69.0
• Physician	39	31.0
Sex		
• Male	26	20.6
• Female	100	79.4
Age		
• 20–25 years	22	17.5
• 26–30 year	56	44.4
• 31–35 years	22	17.5
• 36–40 year	12	9.5
• 41–45 years	1	.8
• 46–50 year	3	2.4
• 51 year and above	10	7.9
Marital Status		
• Married	67	53.2
• Single	50	39.7
• Divorced	1	.8
• Widowed	8	6.3
Qualification		
• Certificate	19	15.1
• Diploma	68	54.0
• First Degree	25	19.8
• Post graduate	14	11.1
Years of service		
• Less than 1 year	25	19.8
• 1–2 years	54	42.8
• 3–4 years	27	21.4
• 5–6 years	6	4.8
• 7–8 years	6	4.8
• 9–10 years	1	.8
• 11 years and above	7	5.6
Unit of Work		
• Antenatal care	26	20.6
• Labour ward	54	42.9
• Lying in ward	14	11.1
• Post natal unit	7	5.6
• All units	25	19.8
Type of Health Facility		
a) Regional hospital	38	30.2
b) District government hospital	42	33.3
➢ Oda hospital	24	19.0
➢ Nsawam hospital	18	14.3
c) District mission hospital	46	36.5
➢ St. Joseph hospital	26	20.6
➢ St. Martin hospital	20	15.9

### Participant’s awareness level, source of information and workshop attendance on MTCT of hepatitis B

From the study, all (100 %, *n* = 126) participants were aware of hepatitis B infection and the main source of information were previous midwifery or medical training school (56.3 %, *n* = 71), followed by workshop/seminar (22.2 %, *n* = 28), and about 14.3 % (*n* = 18) obtained information from more than one source. Also, 64 (50.8 %) of the respondents indicated that they have ever attended a workshop on MTCT of hepatitis B infection as shown in Table 2.

**Table 2:** Summary of table indicating distribution of workshop attendance and source of information on MTCT of HBV among physicians and midwives in the Eastern Region

	Source of information	Frequency	Percentage (%)
Workshop attendance			
❖Yes		64	50.8
❖No		62	49.2
	Workshop/seminar	28	22.2
	During training	71	56.3
	Colleagues	7	5.6
	Mass media	2	1.6
	More than one source	18	14.3

### Knowledge on MTCT of HBV

Participant’s knowledge regarding MTCT of HBV was assessed. In all, 118 (93.7 %) of both physicians and midwives correctly answered the question “hepatitis B infection is caused by virus”. Thirty one percent (31 %) of the participants failed to recognise the need to vaccinate newborns of HBsAg/HBeAg positive mothers before breastfeeding. In addition, only 18 (14.3 %) of the participants knew that hepatitis B surface antigen (HBsAg) is a serological marker for HBV infection. Moreover, only 16 (12.7 %) of the participants knew that there is a vaccine that when administered with hepatitis B Immunoglobulin to newborns of HBV infected mothers can prevent transmission of HBV infection from mother to newborn. Furthermore 45 (35.7 %) considered caesarean section (C/S) as the most effective strategy to prevent mother to child transmission of HBV infection than any available vaccine. Summary of detail response on knowledge of MTCT of HBV is presented in Table 3.

**Table 3:** Response rate and Chi Square test for knowledge on MTCT of HBV infection

Question	Correct		Incorrect	χ^2^
	Midwives	Physician		
Hepatitis B infection is caused by a virus	79 (90.8 %)	39 (100 %)		96.03***
	**118 (93.7 %)**		**8 (6.3 %)**	
Hepatitis B infected mother cannot transmit the hepatitis B infection to her newborn at birth.	84 (96.6 %)	36 (92.3 %)		103.14***
	**120 (95.2 %)**		**6 (4.8 %)**	
It is appropriate for hepatitis B infected mothers (positive HBsAg/HBeAg) to breastfeed their newborn after birth without hepatitis B vaccination.	62 (69.7 %)	25(64.1 %)		18.29***
	**87 (69 %)**		**39(31 %)**	
Unless there is a suspicion, there is no need to screen pregnant women for hepatitis B infection.	79 (90.8 %)	39(100 %)		96.03***
	**118 (93.7 %)**		**8 (6.3 %)**	
Hepatitis B surface antigen (HBsAg) is a serological marker for HBV infection.	15 (17.2 %)	3 (7.7 %)		64.29***
	**18 (14.3 %)**		**108 (85.7 %)**	
So far, there is no vaccine that prevents the transmission of HBV from hepatitis B infected mother to her newborn.	70 (80.5 %)	29 (74.4 %)		41.14***
	**99 (78.6 %)**		**27 (21.4 %)**	
There is a vaccine that when administered with hepatitis B Immunoglobulin to newborns of HBV infected mothers can prevent transmission of the infection from mother to newborn.	12 (13.8 %)	4(10.3 %)		70.13***
	**16 (12.7 %)**		**110 (87.3 %)**	
Caesarean section (C/S) is the most effective strategy to prevent mother to child transmission of HBV infection than any available vaccine.	53 (60.9)	28 (71.8)		10.29**
	**81 (64.3 %)**		**45 (35.7 %)**	
Newborns infected with HBV have no risk of developing liver cancer.	78 (89.7 %)	33 (84.6)		73.14***
	**111 (88.1 %)**		**15 (11.9 %)**	

The bolded frequencies and percentages are the total correct and incorrect response for both midwives and physicians

***p < .001, **p <. 01, *n* = 126, χ^2^ - Chi-square

Knowledge differences on MTCT of HBV were examined among physicians and midwives in regional and district hospitals in the Eastern region of Ghana. In Table 4 above, the result showed no significant differences between physicians (Mean = 6.05, SD = 1.15) and midwives (Mean = 6.11, SD = 1.89) on knowledge on MTCT of HBV [t _(124)_ = .28, *p* = .39]. The average score for knowledge is 6.10 and above. From the result, the mean score for the physicians was below the mean whilst midwives’ was above the mean. However, this difference failed to yield a significant result.

**Table 4:** Summary of mean, standard deviation and Independent *t*-test indicating differences in knowledge on MTCT of HBV between physicians and midwives

	Profession				
	Physicians	Midwives			
	(*N* = 39)	(*N* = 87)	df	t	p
Knowledge on MTCT of HBV	6.05 (1.15)	6.11 (1.89)	124	.28	.39

Standard deviation is presented in parentheses

In terms of type of facility and it differences on knowledge on MTCT of HBV, the result from the One Way ANOVA table (Table 5 above) indicates that at least two of the group means differ from each other [F _(2, 123)_ = 3.69, *p* = .03]. From the multiple comparison analysis, physicians and midwives in the district mission hospitals (Mean = 6.39) had a significant and better knowledge than physicians and midwives in the regional hospital (Mean = 5.71) with a mean difference of 0.68. However, no significant differences were observed between the district mission and government hospitals on Knowledge on MTCT of HBV.

**Table 5:** Summary of mean, standard deviation and one way ANOVA indicating differences in knowledge on MTCT of HBV among type of facility and units of work

Variables	N	Mean	Std. deviation	*df*	*F*	*P*
Type of health facility						
• Regional	38	5.71	.93	2 (123)	3.69*	.03
• District Gov’t	42	6.12	1.02			
• District Mission	46	6.39	1.39			
Units of work						
• ANC	26	6.15	1.22	4 (121)	.23	.92
• Labour ward	54	5.98	1.12			
• Lying in ward	14	6.21	1.31			
• Post natal unit	7	6.14	1.22			
• All units	25	6.20	1.19			

*p is significant at .05 alpha level; df of within group is presented in parentheses

In addition, the result indicated no significant difference among physicians and midwives on knowledge on MTCT of HBV within the different units in the hospitals [F _(4, 121)_ = .23, *p* = .92].

## Discussion

The general objective of this study was to examine the extent of knowledge of physicians and midwives on MTCT of HBV. The findings showed that, all physicians and midwives (100 %) enrolled in the study were aware of HBV infection. This finding is consistent with a 99 % (*n* = 94) HBV awareness level reported in Nigeria among doctors [22]. Perhaps, the recent publicity of the disease in the mass media and other social network in Ghana might have contributed to the increased level of awareness of the disease. Furthermore, the most cited source of HBV-related information in this study was previous medical or midwifery training school (56.3 %). This is lower than a study finding in China which showed that, 71.6 % of obstetric gynaecologist obtained hepatitis B information from their previous medical school [17]. This raises concern since little information on hepatitis B is communicated to student during training [18], coupled with the growing current evidence on viral hepatitis in general in recent times. This may have an implication on Ghana’s response to the burden of HBV since there is a high probability that these critical staff may rely on out-of-date hepatitis B-related information to practice.

More so, continuing professional education is noted to build the capacity of health professionals in terms of knowledge and skills after completion of formal education [23]. However, in this study, 49.2 % of the participants reported that, they have never attended a workshop/seminar on MTCT of HBV since they completed their training. The overall effect is that, these professionals may not be in tune with current evidence that support hepatitis B prevention, management, care and support. Generally, unlike HIV/AIDS which has received much support and funding for capacity building of health professionals [24], less attention has been paid to HBV infection in Ghana. This can be partially attributed to limited local research evidence to support the need for Hepatitis B programmes and few NGOs which champion hepatitis B agenda in the country.

Also, it is widely accepted that, administration of hepatitis B vaccine and immunoglobulin to newborns of positive HBsAg/HBeAg mothers within 12 h after birth is essential to prevent MTCT of HBV [2, 25]. Conversely, this study found that, 87.3 % did not know that there is a vaccine that when administered with hepatitis B Immunoglobulin to newborns of HBV infected mothers can prevent transmission of HBV infection from mother to newborn. This means that, the recommended strategy to prevent MTCT of HBV is not considered primarily by these study participants. A situation that may put babies born to hepatitis B infected mothers to a high risk of acquiring HBV infection especially in Ghana where up to about 16 % of pregnant women are documented to be HBsAg positive [11, 12]. Notwithstanding, CDC recommends that breastfeeding of infant should not be uneccessarily delayed until baby is fully immunised and therefore education can be focused on mothers to ensure that their nipples are devoid of cracks or bleeding [25].

Furthermore, 35.7 % considered caesarean section (C/S) as the most effective strategy to prevent mother to child transmission of HBV infection than any available vaccine. This indicates that, 35.7 % of the participants had a knowledge gap on simple and effective intervention for prevention of MTCT of HBV as recommended by WHO and CDC [2, 25]. This study finding contradict a survey report by Hu and colleagues on knowledge of obstetrics and gynaecology staff which revealed that, majority (86.2 %) of the respondents knew that caesarean section is needless in preventing MTCT of HBV [17]. This therefore suggests the need for education on the effectiveness of hepatitis B vaccine as against caesarean section in preventing HBV from mother-to-child.

This study further revealed that, physicians and midwives in the district mission hospitals showed a good knowledge on MTCT of HBV compared to the regional hospital in the Eastern region. This finding serves as awake up call to focus education on the most appropriate strategies for prevention of MTCT of HBV for physicians and midwives in the study area especially regional government hospital.

### Study limitation

Considering the fact that the study employed a self-report method in data collection, it was difficult to establish the reliable nature of participant’s response. Also, because few hospitals were considered, the study finding is limited to only Eastern region of Ghana but not entire regions of Ghana.

## Conclusion

The outcome of the study showed that, both physicians and midwives on the average had good knowledge on MTCT of hepatitis B infection. However, there were some knowledge gap regarding effective preventive strategies such as the use of hepatitis vaccine and immunoglobulin to prevent mother-to-child transmission of HBV infection. Furthermore, about 49 % of the participants had never attended any workshop on MTCT of hepatitis B since completion of formal training. It is therefore important to build the capacity of health professionals particularly physicians and midwives as part of the strategies to prevent mother to child transmission of hepatitis B in the Eastern region of Ghana.

## Abbreviations

HBsAg, hepatitis B surface antigen; HBV, hepatitis B virus; MTCT, mother-to-child transmission
